# Sprint mechanical variables in elite athletes: Are force-velocity profiles sport specific or individual?

**DOI:** 10.1371/journal.pone.0215551

**Published:** 2019-07-24

**Authors:** Thomas A. Haugen, Felix Breitschädel, Stephen Seiler

**Affiliations:** 1 Norwegian Olympic Federation, Oslo, Norway; 2 Norwegian University of Science and Technology, Trondheim, Norway; 3 Faculty of Health and Sport Sciences, University of Agder, Kristiansand, Norway; Universidade Federal do Rio Grande do Sul, BRAZIL

## Abstract

**Purpose:**

The main aim of this investigation was to quantify differences in sprint mechanical variables across sports and within each sport. Secondary aims were to quantify sex differences and relationships among the variables.

**Methods:**

In this cross-sectional study of elite athletes, 235 women (23 ± 5 y and 65 ± 7 kg) and 431 men (23 ± 4 y and 80 ± 12 kg) from 23 different sports (including 128 medalists from World Championships and/or Olympic Games) were tested in a 40-m sprint at the Norwegian Olympic Training Center between 1995 and 2018. These were pre-existing data from quarterly or semi-annual testing that the athletes performed for training purposes. Anthropometric and speed-time sprint data were used to calculate the theoretical maximal velocity, horizontal force, horizontal power, slope of the force-velocity relationship, maximal ratio of force, and index of force application technique.

**Results:**

Substantial differences in mechanical profiles were observed across sports. Athletes in sports in which sprinting ability is an important predictor of success (e.g., athletics sprinting, jumping and bobsleigh) produced the highest values for most variables, whereas athletes in sports in which sprinting ability is not as important tended to produce substantially lower values. The sex differences ranged from small to large, depending on variable of interest. Although most of the variables were strongly associated with 10- and 40-m sprint time, considerable individual differences in sprint mechanical variables were observed among equally performing athletes.

**Conclusions:**

Our data from a large sample of elite athletes tested under identical conditions provides a holistic picture of the force-velocity-power profile continuum in athletes. The data indicate that sprint mechanical variables are more individual than sport specific. The values presented in this study could be used by coaches to develop interventions that optimize the training stimulus to the individual athlete.

## Introduction

Running a short distance as fast as possible is a core capacity in many sports. For a sprinter competing in athletics, 100 m and 200 m, this capability alone defines them as performers. In bobsleigh, athletes are required to sprint while moving an external mass. Sprinting capacity is also crucial in most team sports, as the ability to either create or close small gaps can be decisive in goal scoring situations. Even in typical endurance sports, explosive acceleration ability (in the context of their slow-twitch dominant peers) can be a medal-winning advantage at the finish of a close race. Accordingly, numerous sprint training studies across a wide range of sports have been performed over the years. Sprinting under assisted, resisted and normal conditions, maximal and explosive strength training, plyometric training and high-intensity running have all been investigated in different combinations [1–3]. Although the principle of specificity is clearly present, no training methods have so far emerged as superior. Individual predispositions must therefore be considered when prescribing training programs.

Considerable effort has been made over the years to quantify the underlying variables for sprint performance. Seminal works by Fenn & Marsh [4] described the force-velocity (Fv) relationship in isolated frog and cat muscles, a relationship that later was confirmed in humans by Wilkie [5]. Advances in technology have allowed scientists to explore fundamental aspects of sprinting skills more closely, and presently, the physiology and mechanics of sprint running are typically interrogated through macroscopic mechanical variables [6–8]. Samozino et al. [9] have recently developed a simple and practical method for profiling the mechanical capabilities of the neuromuscular system using an inverse dynamic approach applied to the centre-of-mass movement, calculating the step-averaged ground reaction forces in runners’ sagittal plane of motion during accelerated sprinting from only anthropometric and spatiotemporal data. Theoretical maximal velocity (v_0_), horizontal force (F_0_), horizontal power (P_max_) and force-velocity profile (i.e., the slope of the force-velocity relationship; S_FV_) can be calculated from the speed-time curve. Other indices, such as ratio of force (RF) and index of force application technique (D_RF_) can also be computed from the same method. RF is a ratio of the step-averaged horizontal component of the ground reaction force to the corresponding resultant force, while D_RF_ expresses the athlete’s ability to maintain a net horizontal force production despite increasing velocity throughout accelerated sprinting [6]. These variables are determinant factors for sprint performance, in line with the laws of motion, and provide insights into individual biomechanical limitations [6–8, 10].

A promising application of force-velocity profiling is in the design of individualized sprint training programs. An effective sprint training program should target the main factors that limit the athlete’s performance [11–12]. To help tailor the training program to the individual, the coach could compare test values from the individual to test values that are typical for the sport. An athlete with a velocity value that is low for the sport could then be prescribed more maximal velocity sprinting, whereas an athlete with a horizontal force value that is low for the sport could be prescribed more horizontal strength training [11]. Currently, sprint force-velocity profile data are only available from athletes in a few selected sports; previous studies only analyzed specialist sprinters or athletes from selected team sports [8, 10, 13–19]. It is unclear whether previously measured sprint force-velocity profiles are specific to the sport or specific to the athlete. To individualize a training program for an athlete in a given sport, the coach requires a holistic picture of the force-velocity profile continuum in athletes. Therefore, the aim of the present study was to quantify differences in sprint mechanical variables across many sports and within each sport. Secondary aims were to quantify sex differences and relationships among the variables.

## Materials and methods

### Participants

The Norwegian Olympic training centre is a standardized testing facility used by a large number of elite athletes from many sports. A database of results from 40-m sprint tests was collected from 1995 to 2018, and this database provides a foundation for exploring sprint performance and mechanical properties in athletes. In this cross-sectional study we analyzed sprint tests by 666 athletes from 23 sports. All participants were Norwegian national team athletes, i.e., represented Norway in international senior competitions, and 128 of the athletes were medalists from the World Championships and/or Olympic Games.

### Ethics statement

This study was based on pre-existing data from quarterly or semi-annual testing that the athletes performed for training purposes, and thus no informed consent was obtained [20]. All data were anonymized to comply with the General Data Protection Regulations of the European Union. The study was reviewed by the Norwegian Data Protection Authority and approved by the ethics committee at the Faculty of Health and Sport Sciences, University of Agder (reference number 19/00068).

### Procedures and data handling

All included players were tested in the time period 1995–2018. Tests were performed on a dedicated indoor 40-m track with 8 mm Mondotrack FTS surface (Mondo, Conshohocken, USA) at the Norwegian Olympic training center in Oslo. A standard warm-up program was completed prior to sprint testing, beginning with a 10–15 min easy jog, followed by 5–6 minutes of sprint specific drill exercises, 2–4 strides with increasing speed and 1–2 trial starts. During testing, athletes assumed the starting position and started running on their own initiative after being cleared to start by the test leader. New trials were performed every 3–5 min until a performance plateau was observed. In practice, 80% of all players achieved their best performance within two trials. Body mass was assessed immediately prior to or after the sprint test on a stationary force platform (AMTI, model OR6-5-1). Data from a single athlete was only included in one category for each analysis. That category was the player’s affiliation on the day of his/her best sprint test result. A purpose-built excel spreadsheet developed by Morin & Samozino [21] formed basis for calculations of F_0_, v_0_, P_max_, S_FV_, RF_max_ and D_RF_. These calculations were based on best individual sprint test, associated split times and body mass. Temperature and atmospheric pressure were set to 760 mm Hg and 20 °C.

Two different timing system setups were used. In the time epoch 1995–2011, a 60x60 cm start pad was placed under the track at the start line. The clock was initiated when the front foot stepped off the pad. Split times were recorded with split-beamed timing gates for each 10^th^ m of the sprints. The transmitters where placed 140 cm above ground level, while the reflectors were placed 130 and 150 cm above the floor. Both beams had to be interrupted to trigger each timing gate. The timing setup has been assessed for accuracy and reliability [22]. In January 2011, the timing system was upgraded. The split-beamed timing gates were replaced by dual-beamed timing gates, while the start pad was replaced with a single-beamed timing gate located 60 cm in front of the start line and 50 cm above ground level. Rigorous pilot testing was performed before deciding the exact location of the timing gate at start to provide comparable times with the previous setup. Simultaneous comparisons (*n* = 50) of the old and new timing setup revealed no differences in 40-m sprint times (mean ± SD: 0.00 ± 0.02 s). The dual-beamed timing system has also been assessed for accuracy and reliability [23]. Overall, typical error (coefficient of variation; CV) was 0.6–2.4% for sprint times, ~1.5% for v_0_ and RF_max_, and 3.5–5.1% for F_0_, P_max_, S_FV_ and D_RF_ for both timing setups.

To ensure valid sprint mechanical values when using split times as input in the method proposed by Samozino et al. [9], it is crucial that i) the entire acceleration phase is captured, and ii) time initiation (the time 0) is very close to the first rise of the force production onto the ground [24]. For the current procedures, the body’s center-of-mass was ~ 0.5 m in front of the start line, and possessed a considerable forward momentum, at time triggering. Hence, based on available correction factors [22, 25], 0.5 s was added to all sprint times for converting to “first movement” triggering. All sprint times presented are comparable to starts from blocks and audio signal with reaction time subtracted from the total time.

### Statistical analysis

Data are reported as mean ± SD. Pearson’s R (±90%CL) was used to examine the relationship across variables (after log transformation of physiological data). Correlation values were interpreted categorically according to the scale outlined by Hopkins et al. [26], meaning that 0.10, 0.30, 0.50, 0.70, 0.90 and 1.0 were thresholds for small, moderate, large, very large, extremely large and perfect, respectively. Magnitudes of differences across category means were assessed by standardization (mean difference divided by the harmonic mean of the SD of the compared groups). The thresholds for assessing the observed difference in means were 0.2, 0.6, 1.2, 2.0 and 4.0 for small, moderate, large, very large and extremely large, respectively [26]. To make inferences about true values of effects, non-clinical magnitude-based inference rather than null-hypothesis significance testing was used [26, 27]. Magnitudes were evaluated mechanistically: if the confidence interval overlapped substantial positive and negative values, the effect was deemed unclear; otherwise effects were deemed clear and shown with the probability that the true effect was substantial using the following scale: 25–75%, possibly; 75–95%, likely; 95–99.5%, very likely; > 99.5%, most likely [26, 27]. A purpose-built excel spreadsheet for combining outcomes from several subject groups was used to calculate effect magnitudes, confidence limits (CL) and inferences [28]. Women’s performance was defined as 100% for comparisons between male and female athletes (all sport disciplines pooled together).

## Results

To keep the results within reasonable limits, only a summary of the results is presented in this section. However, additional comparisons across category means can be performed by inserting data from the supplementary file into Hopkins’ spreadsheet [28].

Table 1 shows age, body mass and sprint performance across the analyzed sports. Overall, athletics sprinting (hereafter referred to as “sprinting” or “sprinters”) produced the fastest sprint times over both the shortest (10 m) and longest (40 m) distances among males, clearly ahead of bobsleigh (mean difference, ±90%CL: 0.02, ±0.03 and 0.09, ±0.09 s; possibly and likely; moderate), athletics jumping (hereafter referred to as “jumping” or “jumpers”) (0.07, ±0.03 and 0.15, ±0.09 s; most likely and very likely; large), soccer (0.11, ±0.02 and 0.38, ±0.07 s; most likely; very large) and all other sports (0.13, ±0.03 to 0.24, ±0.03 and 0.45, ±0.09 to 0.88, ±0.09 s; most likely; very large to extremely large). Sprinters also displayed the fastest sprint times over the same distances among women, clearly ahead of jumpers (0.10, ±0.04 and 0.28, ±0.15 s; most likely and very likely; very large), handball (0.15, ±0.03 and 0.52, ±0.11 s; most likely; very large), athletics throwing (0.16, ±0.04 and 0.57, ±0.13 s; most likely; very large) and all other sports (0.17, ±0.02 to 0.34, ±0.05 and 0.61, ±0.13 to 1.23, ±0.25 s; most likely; very large to extremely large). The mean sex difference increased from 6.4% for 10-m sprints to 9.3% for 40-m sprints.

**Table 1:** Mean values (± SD) of age, body mass and sprint performance in Norwegian national team athletes (*n* = 666).

Discipline (sex)	*N*	Age	BM	10 m	20 m	30 m	40 m
		*(y)*	*(kg)*	*(s)*	*(s)*	*(s)*	*(s)*
Alpine skiing (W)	10	22.6 ± 3.3	66.0 ± 4.2	2.19 ± 0.05	3.62 ± 0.10	4.95 ± 0.15	6.29 ± 0.19
Alpine skiing (M)	13	24.7 ± 3.8	84.4 ± 3.7	2.04 ± 0.05	3.30 ± 0.08	4.48 ± 0.10	5.64 ± 0.13
Athletics jumping (W)	8	20.4 ± 4.9	60.4 ± 2.6	2.10 ± 0.06	3.40 ± 0.10	4.60 ± 0.15	5.79 ± 0.19
Athletics jumping (M)	9	21.8 ± 3.6	77.4 ± 6.8	1.97 ± 0.05	3.16 ± 0.06	4.23 ± 0.09	5.28 ± 0.11
Athletics sprinting (W)	5	19.2 ± 3.0	58.4 ± 2.1	2.00 ± 0.02	3.24 ± 0.04	4.39 ± 0.05	5.51 ± 0.09
Athletics sprinting (M)	8	20.7 ± 3.2	71.8 ± 3.8	1.90 ± 0.03	3.05 ± 0.05	4.09 ± 0.08	5.13 ± 0.11
Athletics throwing (W)	10	20.3 ± 3.6	75.0 ± 6.6	2.16 ± 0.06	3.53 ± 0.09	4.81 ± 0.14	6.08 ± 0.20
Athletics throwing (M)	14	22.4 ± 4.7	100.1 ± 8.8	2.03 ± 0.07	3.30 ± 0.11	4.45 ± 0.15	5.58 ± 0.20
Bandy (W)	13	23.0 ± 5.4	64.1 ± 8.6	2.28 ± 0.07	3.76 ± 0.12	5.18 ± 0.16	6.63 ± 0.21
Bandy (M)	23	19.3 ± 2.7	77.4 ± 8.3	2.09 ± 0.06	3.39 ± 0.09	4.60 ± 0.12	5.80 ± 0.16
Basketball (M)	10	22.6 ± 3.3	86.2 ± 9.5	2.06 ± 0.07	3.36 ± 0.12	4.55 ± 0.17	5.74 ± 0.22
Beach-/volleyball (M)	23	24.9 ± 4.7	87.7 ± 7.7	2.04 ± 0.05	3.34 ± 0.09	4.56 ± 0.13	5.78 ± 0.16
Bobsleigh (M)	9	26.7 ± 1.9	92.8 ± 4.4	1.92 ± 0.03	3.10 ± 0.06	4.17 ± 0.07	5.22 ± 0.09
Combat sports (W)	17	23.7 ± 6.0	60.6 ± 5.7	2.30 ± 0.07	3.78 ± 0.12	5.20 ± 0.19	6.62 ± 0.26
Combat sports (M)	32	22.5 ± 4.2	74.8 ± 11.1	2.08 ± 0.07	3.38 ± 0.11	4.59 ± 0.16	5.80 ± 0.23
Cross-country skiing (W)	8	20.0 ± 3.4	59.9 ± 5.1	2.27 ± 0.10	3.73 ± 0.19	5.12 ± 0.28	6.51 ± 0.37
Cross-country skiing (M)	15	21.9 ± 3.4	74.2 ± 4.4	2.11 ± 0.10	3.44 ± 0.16	4.68 ± 0.23	5.88 ± 0.31
Fencing (W)	5	18.9 ± 1.0	64.6 ± 4.0	2.34 ± 0.06	3.86 ± 0.11	5.30 ± 0.18	6.74 ± 0.25
Fencing (M)	10	21.9 ± 2.0	77.1 ± 6.6	2.14 ± 0.04	3.50 ± 0.07	4.76 ± 0.10	6.01 ± 0.13
Handball (W)	32	25.8 ± 4.6	72.8 ± 6.1	2.15 ± 0.07	3.50 ± 0.13	4.77 ± 0.18	6.03 ± 0.24
Handball (M)	18	23.9 ± 3.6	92.7 ± 9.0	2.03 ± 0.04	3.27 ± 0.07	4.43 ± 0.10	5.58 ± 0.14
Ice hockey (M)	34	24.8 ± 4.6	88.7 ± 7.4	2.03 ± 0.06	3.30 ± 0.10	4.46 ± 0.14	5.62 ± 0.19
Mogul skiing (W)	5	19.4 ± 1.9	64.0 ± 9.1	2.18 ± 0.04	3.57 ± 0.10	4.88 ± 0.16	6.19 ± 0.22
Mogul skiing (M)	14	21.2 ± 3.1	72.9 ± 6.3	2.05 ± 0.04	3.32 ± 0.05	4.52 ± 0.09	5.67 ± 0.12
Nordic combined (M)	22	23.5 ± 4.2	69.6 ± 4.0	2.04 ± 0.05	3.33 ± 0.08	4.51 ± 0.11	5.69 ± 0.16
Ski jumping (W)	11	18.4 ± 4.1	56.3 ± 3.1	2.23 ± 0.04	3.68 ± 0.06	5.05 ± 0.11	6.45 ± 0.15
Ski jumping (M)	28	21.2 ± 3.5	64.3 ± 5.0	2.05 ± 0.07	3.34 ± 0.12	4.55 ± 0.18	5.75 ± 0.25
Snowboard (W)	5	21.7 ± 4.6	59.4 ± 3.4	2.24 ± 0.06	3.73 ± 0.11	5.13 ± 0.17	6.61 ± 0.24
Snowboard (M)	9	21.3 ± 3.1	78.5 ± 7.7	2.05 ± 0.05	3.34 ± 0.09	4.55 ± 0.11	5.76 ± 0.16
Soccer (W)	93	23.8 ± 3.9	64.0 ± 4.9	2.17 ± 0.06	3.55 ± 0.11	4.84 ± 0.16	6.12 ± 0.22
Soccer (M)	57	25.4 ± 4.3	78.7 ± 5.8	2.01 ± 0.05	3.24 ± 0.08	4.39 ± 0.12	5.51 ± 0.16
Speed skating (W)	12	21.4 ± 4.0	68.5 ± 5.8	2.20 ± 0.05	3.60 ± 0.08	4.92 ± 0.12	6.25 ± 0.17
Speed skating (M)	22	22.3 ± 3.7	78.6 ± 8.0	2.10 ± 0.06	3.39 ± 0.12	4.59 ± 0.17	5.78 ± 0.23
Table tennis (M)	13	21.1 ± 4.1	69.5 ± 8.8	2.12 ± 0.06	3.46 ± 0.13	4.71 ± 0.19	5.95 ± 0.27
Telemark skiing (W)	5	18.6 ± 1.7	62.0 ± 1.9	2.26 ± 0.13	3.72 ± 0.23	5.10 ± 0.35	6.49 ± 0.49
Telemark skiing (M)	13	23.3 ± 2.6	82.7 ± 6.3	2.08 ± 0.06	3.39 ± 0.08	4.60 ± 0.11	5.80 ± 0.14
Tennis (W)	7	17.5 ± 1.7	65.6 ± 3.3	2.25 ± 0.07	3.70 ± 0.12	5.08 ± 0.18	6.48 ± 0.24
Tennis (M)	11	20.8 ± 3.3	75.3 ± 4.8	2.07 ± 0.05	3.37 ± 0.07	4.57 ± 0.10	5.78 ± 0.13
Weight-/powerlifting (M)	13	20.6 ± 4.2	87.9 ± 22.2	2.12 ± 0.07	3.45 ± 0.10	4.71 ± 0.17	5.96 ± 0.22

W = women, M = men, BM = body mass.

**Fig 1:**
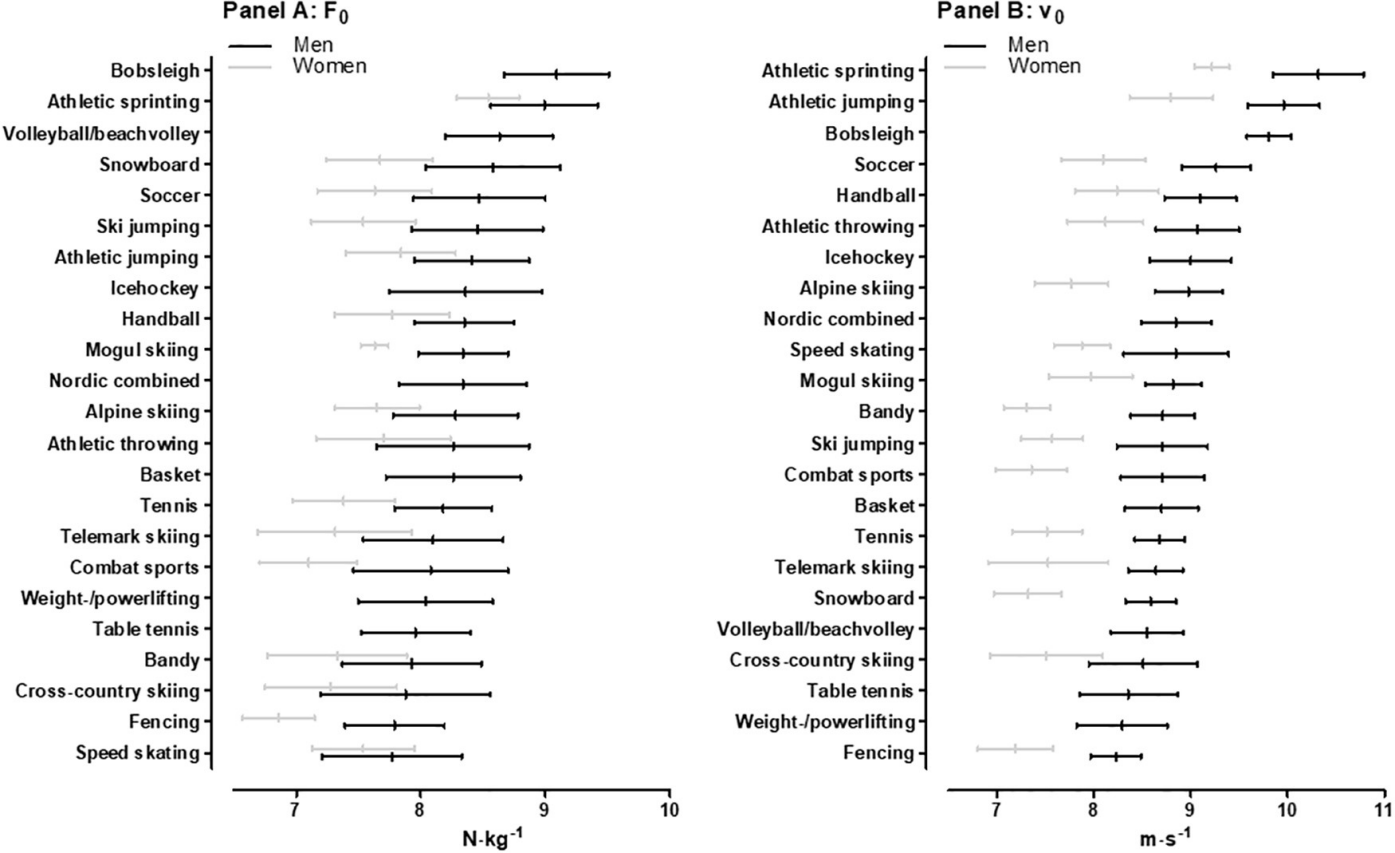
Maximal horizontal force (F_0_) (Panel A) and theoretical maximal velocity (v_0_) (Panel B) across sports. The sports are ranked according to mean values for men.

Fig 1A shows F_0_ across sports. Bobsleigh and sprinting displayed the greatest F_0_ values among men (unclear difference between them), clearly ahead of volleyball/beach volleyball (0.4, ±0.3 to 0.5, ±0.3 N·kg^-1^; likely to very likely; moderate), snowboard (0.4, ±0.4 to 0.5, ±0.4 N·kg^-1^; likely to very likely; moderate), soccer (0.5, ±0.3 to 0.6, ±0.3 N·kg^-1^; very likely to most likely; moderate to large) and all other sports (0.5, ±0.3 to 1.3, ±0.3; very likely to most likely; moderate to very large). In women, sprinters exhibited the highest F_0_ values, clearly ahead of jumping (0.7, ±0.4 N·kg^-1^; most likely; very large), handball (0.8, ±0.3 N·kg^-1^; most likely; very large), snowboard (0.9, ±0.4 N·kg^-1^; very likely; very large), alpine skiing (0.9, ±0.3 N·kg^-1^; most likely; very large) and all other sports (0.9, ±0.3 N·kg^-1^ to 1.7, ±0.3 N·kg^-1^; most likely; very large to extremely large). The sex difference for F_0_ was 9.3, ±1.2% (most likely; moderate).

Fig 1B shows v_0_ across sports. Sprinters showed the highest values among men, clearly ahead of jumpers (0.4, ±0.4 m·s^-1^; likely; moderate), bobsleigh (0.5, ±0.3 m·s^-1^; very likely; large), soccer (1.1, ±0.3 m·s^-1^; most likely; very large) and all other male sports (1.2, ±0.3 to 2.1, ±0.3 m·s^-1^; most likely; very large to extremely large). Sprinters also displayed superior values among women, clearly better than jumpers (0.4, ±0.3 m·s^-1^; very likely; moderate), handball (1.0, ±0.2 m·s^-1^; most likely; very large), athletics throwing (1.1, ±0.2 m·s^-1^; most likely; very large) and all other female sports (1.1, ±0.2 to 2.0, ±0.4 m·s^-1^; most likely; very large to extremely large). The sex difference for v_0_ was 11.9, ±1.1% (most likely; large).

**Fig 2:**
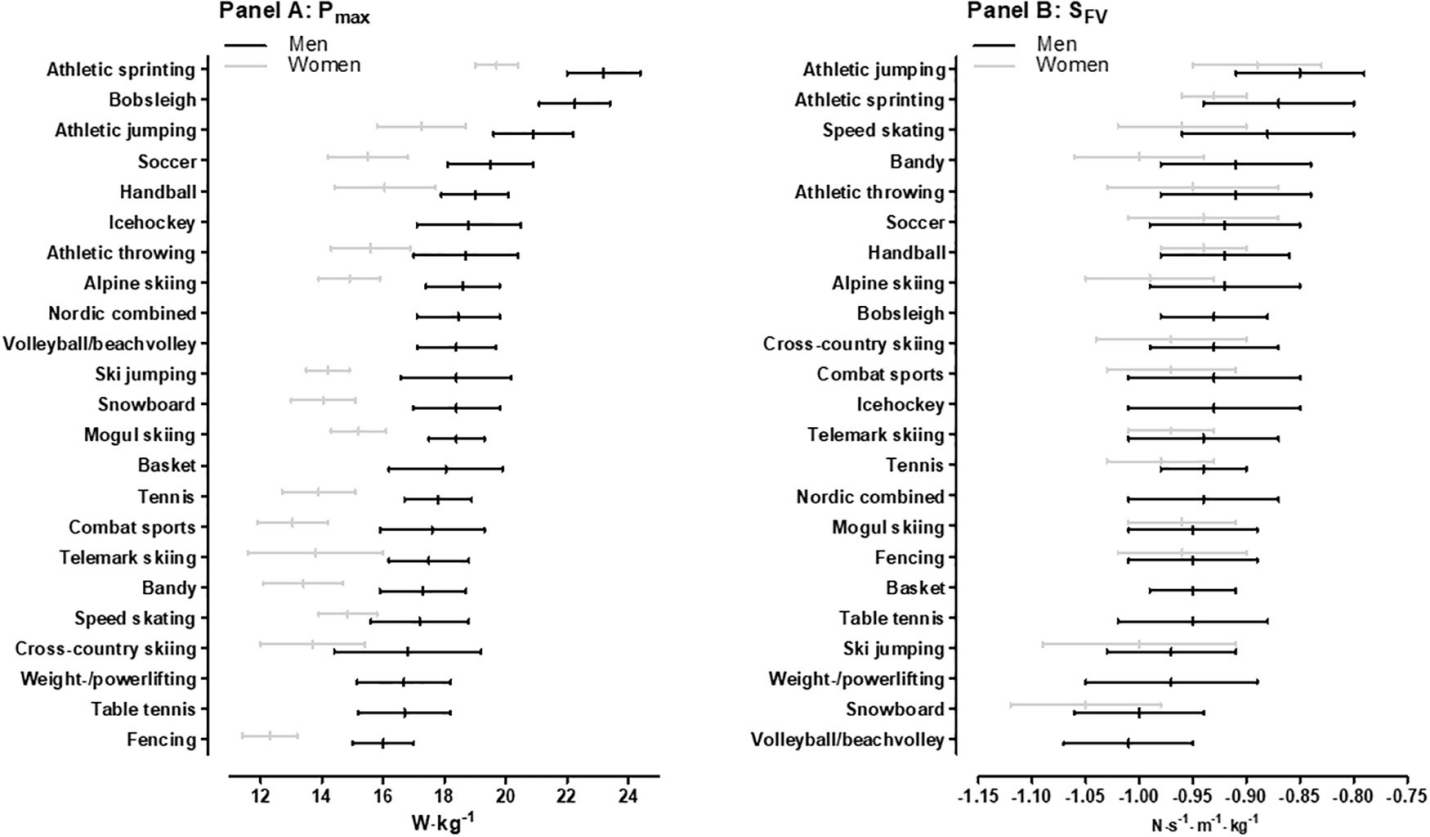
Maximal horizontal power (P_max_) (Panel A) and force-velocity slope (S_FV_) (Panel B) across sports. The sports are ranked according to mean values for men.

Fig 2A shows P_max_ across sports. In men, sprinters obtained the highest values, clearly ahead of bobsleigh (0.9, ±1.0 W·kg^-1^; likely; moderate), jumping (2.2, ±1.1 W·kg^-1^; most likely; large), soccer (3.7, ±0.8 W·kg^-1^; most likely; very large) and all other male sports (4.2, ±0.9 to 7.2, ±0.9 W·kg^-1^; most likely; very large to extremely large). Sprinting athletes also displayed the highest P_max_ values among women, clearly ahead of jumping (2.4, ±1.1 W·kg^-1^; most likely; very large), handball (3.6, ±0.8 W·kg^-1^; most likely; very large), throwers (4.0, ±0.9 W·kg^-1^; most likely; extremely large) and all other female sports (4.2, ±0.7 to 7.3, ±0.8 W·kg^-1^; most likely; extremely large). The sex difference for P_max_ was 21.9, ±1.1% (most likely; large).

Fig 2B shows S_FV_ across sports. Jumpers produced the highest values (i.e., most velocity-oriented) among the males, ahead of sprinting specialists (0.02, ±0.04 N·s^-1^·m^-1^·kg^-1^; unclear; small), speed skating (0.03, ±0.04 N·s^-1^·m^-1^·kg^-1^; possibly; small) and all other male sports (0.06, ±0.04 to 0.16, ±0.03 N·s^-1^·m^-1^·kg^-1^; very likely to most likely; moderate to very large. At the other end, volleyball/beach volleyball and snowboard were the most force-oriented disciplines (unclear difference between them), showing clearly lower S_FV_ values than weight-/powerlifting (0.03, ±0.04 to 0.04, ±0.04 N·s^-1^·m^-1^·kg^-1^; likely: small to moderate) and all other male sports (0.03, ±0.04 to 0.16, ±0.03 N·s^-1^·m^-1^·kg^-1^; likely to most likely; small to very large). Among female athletes, jumpers displayed the most velocity-based S_FV_ values, clearly higher than athletic sprinting (0.04, ±0.05 N·s^-1^·m^-1^·kg^-1^; likely; moderate) and all other female sports (0.05, ±0.04 to 0.16, ±0.08 N·s^-1^·m^-1^·kg^-1^; likely to most likely; moderate to very large). Snowboard was the most force-oriented group, ahead of bandy (0.05, ±0.07 N·s^-1^·m^-1^·kg^-1^; unclear; moderate), ski jumping (0.05, ±0.08 N·s^-1^·m^-1^·kg^-1^; unclear; moderate) and all other female sports (0.06, ±0.07 to 0.16, ±0.08 N·s^-1^·m^-1^·kg^-1^; likely to most likely; moderate to very large). The sex difference for S_FV_ was 2.4, ±0.7% (most likely; small).

**Fig 3:**
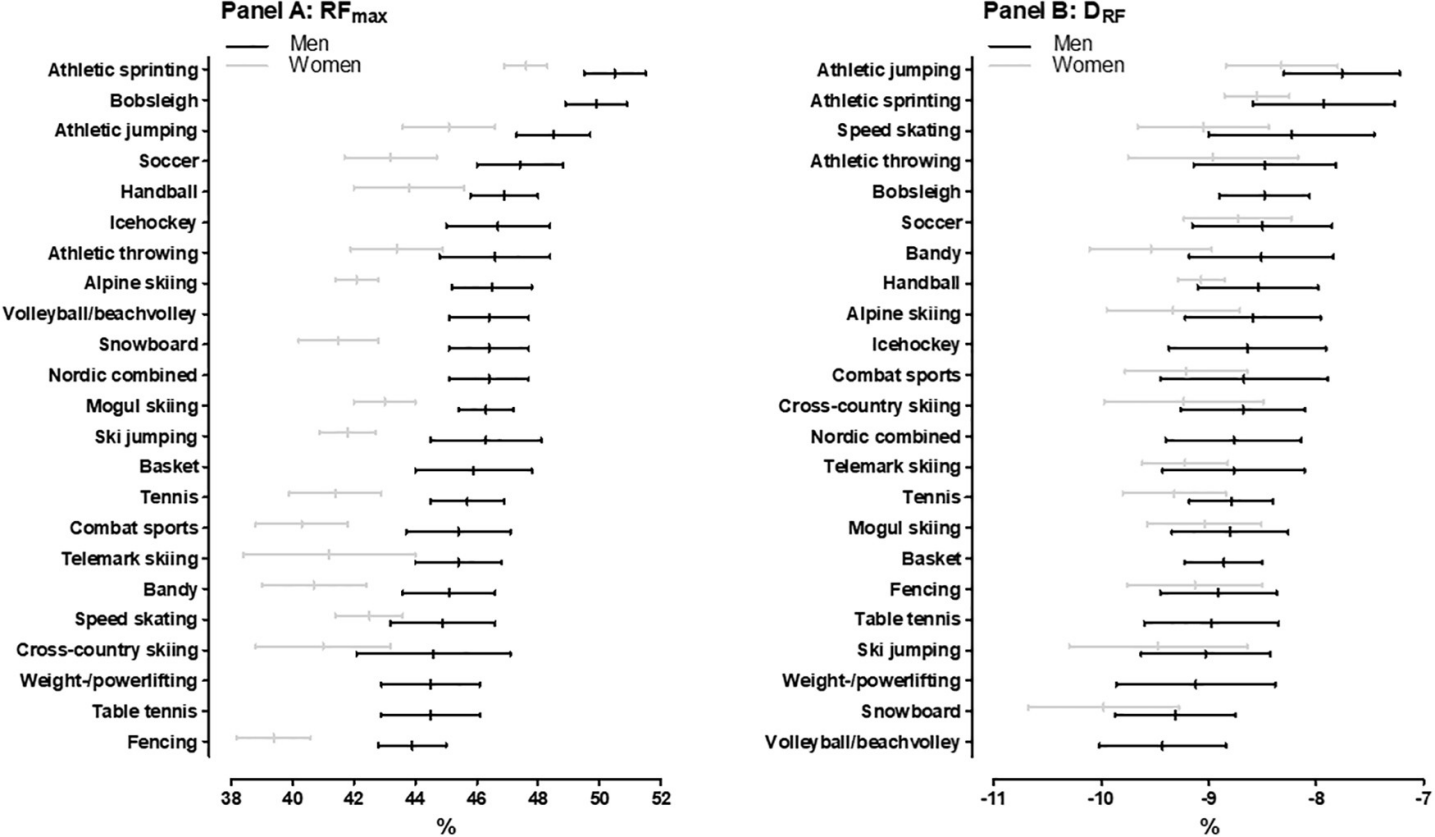
Maximal ratio of force (RF_max_) (Panel A) and index of force application technique (D_RF_) (Panel B) across sports. The sports are ranked according to mean values for men.

Fig 3A shows RF_max_ across sports. Sprinters produced the highest percentage values among men, ahead of bobsleigh (0.4, ±0.8%; unclear; small), jumping (1.4, ±0.8%; very likely; large) and all other male sports (2.1, ±0.7 to 4.6, ±0.7%; most likely; large to extremely large). Sprinters also displayed the highest values among women, clearly ahead of jumpers (1.7, ±1.1%; very likely; large) and all other sports (2.9, ±0.9 to 5.9, ±1.2%; most likely; very large to extremely large).

Fig 3B shows D_RF_ across sports. Jumpers displayed the highest values, ahead of sprinters (0.2, ±0.4%; unclear; small), speed skating (0.4, ±0.4%; likely; moderate) and all other male sports (0.7, ±0.4 to 1.5, ±0.3%; very likely to most likely; moderate to very large). Among females, jumpers also obtained the highest values, clearly ahead of sprinters (0.2, ±0.4%; unclear; small), handball (0.5, ±0.3%; very likely; moderate) and all other female sports (0.5, ±0.3 to 1.5, ±0.7%; very likely to most likely; moderate to very large).

Table 2 shows correlations (±90%CL) across the analyzed variables. The correlations between sprint mechanical variables and sprint times ranged from trivial to perfect. The correlations between sprint performance and S_FV_/D_RF_ increased with increasing sprint distance.

**Table 2:** Correlations (±90%CL) across analyzed variables.

	**10-m time**	**40-m time**	**F_0_**	**v_0_**	**P_max_**	**S_FV_**	**RF_max_**
**40-m time**	0.96, ±0.01						
**F**_**0**_	-0.89, ±0.01	-0.73, ±0.03					
**v**_**0**_	-0.87, ±0.02	-0.97, ±0.01	0.57, ±0.04				
**P**_**max**_	-1.00, ±0.01	-0.97, ±0.01	0.88, ±0.02	0.89, ±0.01			
**S**_**FV**_	-0.02, ±0.06	-0.30, ±0.06	-0.43, ±0.05	0.50, ±0.05	0.05, ±0.06		
**RF**_**max**_	-1.00, ±0.01	-0.96, ±0.01	0.89, ±0.01	0.88, ±0.02	1.00, ±0.01	0.03, ±0.06	
**D**_**RF**_	-0.15, ±0.06	-0.42, ±0.05	-0.30, ±0.06	0.62, ±0.04	0.19, ±0.06	0.99, ±0.01	0.17, ±0.06

F_0_ = maximal horizontal force (relative to body mass), v_0_ = theoretical maximal velocity, P_max_ = maximal horizontal power (relative to body mass), S_FV_ = force-velocity slope (relative to body mass), RF_max_ = ratio of force, D_RF_ = index of force application technique.

## Discussion

To our knowledge, this is the first study to explore and compare underlying physiological and mechanical variables of sprint performance across a wide range of sports. Up to very large and even extremely large differences in sprint mechanical variables were observed across sports. Overall, sports in which sprinting ability is an important predictor of success scored the highest values for most variables, while sports involving other locomotion modalities than running tended to produce substantially lower values. The current data from a large sample of elite athletes tested under identical conditions provides a holistic picture of the Fv profile continuum in sprinting athletes. In the following paragraphs, we will discuss each of the analysed variables more in detail.

F_0_ in the present sample was in the range 7–10 N∙kg^-1^ for men and 6–9 N∙kg^-1^ for women, corresponding to a mean sex difference of 9.3%. Athletic sprinters and bobsleigh contestants were at the upper end of the scale. Previously published studies have shown that world-class male and female sprinters can reach 11 and 10 N∙kg^-1^, respectively [10], representing the current upper limits for horizontal force production relative to body mass during accelerated sprinting. However, F_0_, calculated with the simple method outlined by Samozino et al. [9], is larger during resisted sprinting compared to unloaded sprints [14, 15, 29]. Hence, F_0_ derived from normal sprinting appears not to be a true F_0_, as the resistance in overcoming body mass inertia appears insufficient for maximal horizontal force-capacity generation. For practitioners, the importance of F_0_ is perfectly illustrated by the fact that bobsleigh athletes displayed slightly higher F_0_-values (and clearly higher body mass) than sprinters despite slightly poorer sprint times across all time splits. Thus, F_0_ is a particularly crucial measure for athletes who perform brief sprints while moving an external mass. The shorter the distance considered, the higher the correlation between sprint performance and F_0_ (see Table 2).

Cross et al. [17] reported 8.5 ± 1.3 and 8.8 ± 0.4 N∙kg^-1^ for elite rugby union forwards and backs, and 8.1 ± 0.8 and 8.2 ± 1.0 N∙kg^-1^ for corresponding rugby league players. These F_0_-values are on par with the present male soccer players. Interestingly, although rugby players are generally heavier than soccer players, they do not produce higher F_0_ when normalized for body mass. As an athlete gets heavier, the energy cost of accelerating that mass also increases, as does the aerodynamic drag associated with pushing that wider frontal area through the air [30]. “Bigger” is therefore not necessarily better for sprinting, at least when there is no external mass to push. Moreover, volleyball/beach volleyball were among the best sports in terms of F_0_ scores, while weight-/powerlifters produced clearly lower values, despite no substantial group mean differences in body mass. This confirms previous findings that vertically-oriented and heavy strength training of the lower limbs does not necessarily translate to higher horizontal force production during accelerated sprinting [31].

The correlation between v_0_ and sprint performance was very large for 10-m sprint, and the correlation values increased with increasing sprint distance (see Table 2). V_0_ was in the range 7.5–11 m∙s^-1^ for men and 6–9.5 m∙s^-1^ for women, equivalent to a mean sex difference of 11.9%. Not surprisingly, sprinters obtained the highest scores. For comparison, the world’s fastest male and female track sprinters reach peak velocities of ~12 and ~11 m·s^-1^, respectively [10, 32]. The fastest male and female team sport athletes in our material approached/exceeded 10 and 9 m∙s^-1^, respectively, in line with previous reports [33–35]. Many practitioners would argue that elite wide receivers, running backs and/or cornerbacks in American football are even faster, but no studies to date have presented comparable data.

Metabolic energy turnover and efficient transfer to external power output underlies successful performance in many sports. We observed perfect or extremely large correlations between P_max_ and sprint performance, depending on distance (Table 2). Sprint time improvement is not a linear function of power increase. Indeed, the change in velocity (Δv) is related to the cube of the change in power (ΔP^3^), such that a 5% increase in velocity would require a nearly 16% increase in power [36]. The present data are consistent with this relationship. We observed P_max_ values in the range 13–25 W∙kg^-1^ for men and 11–21 W∙kg^-1^ for women. The mean sex difference observed (21.9%), based on pooled data of all disciplines, corresponds well with data for elite sprinters. Slawinski et al. [10] reported that P_max_ in male and female world-class sprinters was 30.3 ± 2.5 and 24.5 ± 4.2 W·kg^-1^, respectively, typically attained after ~1 s of sprinting. The highest individual values were 36.1 W·kg^-1^ and 29.3 W·kg^-1^, representing current upper limits in humans [37]. Athletes achieve two-to-three times higher W·kg^-1^ during countermovement jump (CMJ) compared to sprinting [37]. However, because it is not possible to assess a generic anaerobic maximal power, each anaerobic power test must be treated separately, and comparisons of power values across modalities are meaningless.

S_FV_ reflects the athlete’s individual balance between force and velocity capabilities. Morin & Samozino [11] have suggested that athletes with velocity values lower for the sport could be prescribed more maximal velocity sprinting, whereas athletes with horizontal force values lower for the sport could be prescribed more horizontal strength training. The present results show that S_FV_ ranged from -0.75 to -1.10 N·s^-1^·m^-1^·kg^-1^ for men and -0.80 to -1.15 N·s^-1^·m^-1^·kg^-1^ for women, corresponding to a sex difference of 2.4%. The group mean values of national-level sprinters were similar to the S_FV_ values observed in world-class sprinters [10]. In the current study, women displayed lower slope values than men in all the analyzed sports where both sexes were represented. If we follow the approach proposed by Morin & Samozino [11], men should generally perform more force-oriented training than women. To our knowledge, no previous studies have recommended differentiated sprint-training programs according to sex. While young and untrained individuals tend to show improvements irrespective of training methods [38], well-trained senior athletes only achieve annual improvements smaller than typical variation [39].

Sprinting distance must also be considered if training prescription should be based on S_FV_ orientation. The correlation between sprint performance and S_FV_ increased (towards more velocity-oriented FV-profile) with increasing sprint distance (Table 2). This suggests that the longer the sprint distance, the more velocity-oriented training should be prescribed. Force-oriented sprint training (e.g., resisted sprints) is likely more appropriate for sports where the athletes are required to perform brief sprints while moving an external mass (e.g., bobsleigh). However, macroscopic Fv profiles derived from the simple method provide limited information about the Fv relationship of the individual muscles involved. The fascicle shortening velocity of the different muscles engaged do not necessarily change with increasing running velocity, and this inconsistent relationship is explained by an augmented contribution from elastic properties with increasing running velocity [40, 41]. Hence, running velocity is not a proxy for muscle contraction velocity, and one cannot use Fv profiles derived from sprint tests to determine training prescriptions for muscles in isolation.

RF_max_ reflects the proportion of the total force production that is directed in the forward direction of motion at sprint start [9]. We observed a perfect correlation between RF_max_ and P_max_ (*r* = 1.0, Table 2). This is mechanically sound, as P_max_ in the simple model corresponds to the peak of the power curve (i.e., the maximal product of horizontal force and velocity), while vertical force corresponds to body mass when averaged over one step. Hence, P_max_ and RF_max_ are two measures of the same capability. Within our material, RF_max_ ranged from 41 to 52% in men and 37 to 48% in women. Rabita et al. [8] reported 71.6 ± 2.6% in male world-class sprinters, but these values are not directly comparable due to methodological differences. In the Rabita et al. paper [8], RF was computed from force platform data, where the y-intercept of the extrapolated linear RF-velocity curve was defined as RF_max_, that is; the theoretical maximal contribution of anteroposterior force to the total force produced over one contact phase at zero velocity. For the simple method, where mechanical variables are calculated from anthropometric and spatiotemporal data, RF_max_ does not correspond to the extrapolated value at zero velocity, but to the value at 0.5 s (corresponding to the RF-value approximately at the first step). Based on publicly available split time and anthropometric data of world-class sprinters [42], we calculated RF_max_ values at 0.5 s as high as 56–57% in men and 52–53% in women, using the simple model. However, the maximal possible value of RF_max_ (100%) is not optimal for sprinting because a certain amount of vertical force is required to work against gravity.

D_RF_ expresses the athletes’ ability to maintain a net horizontal force production despite increasing running velocity. The more negative the slope, the faster the loss of net horizontal force during acceleration, and vice versa. In the present dataset, the values ranged from -7 to -10.5% among the men and -7.5 to -11% among the women. For comparisons, Rabita et al. [8] reported −6.4 ± 0.3% for male world-class sprinters. In practical terms, D_RF_ reflects the distance over which athletes are able to accelerate (i.e., distance to peak velocity). Previous research has shown that the duration of the acceleration phase varies as a function of athlete performance level. Team sport athletes [33, 34], students [43] and prepubescent children [44] typically achieve peak velocity at ~ 25–30 m of maximal linear sprinting. National and international 100-m track sprinters attain peak velocity after 40–50 and 50–80 m of sprinting, respectively, but men peak ~20% further in distance than women [10, 32, 45]. The nearly perfect correlation between D_RF_ and S_FV_ (*r* = 0.99, Table 2) is logical and expected, as the most velocity-oriented athletes are able to accelerate over a longer distance than their more force-oriented counterparts.

## Conclusion

In the present study, substantial differences in sprint mechanical properties were observed across sports. Based on these findings, some may argue that the chronic practice of an activity induces different Fv profiles in sprint running over time. However, the large spread within each discipline, in addition to the large overlap across sports, indicate that such variables are more individual than sport specific. Most sprint mechanical variables are strongly correlated with sprint performance level, in line with the laws of motion. Indeed, when split times and anthropometric data form basis for calculations of multiple variables, it is reasonable to expect high correlations among them. Based on these considerations, practitioners may question the practical relevance of such variables, as they are entwined, and in some cases, mere ‘different explanations of the same story’. However, while split time data provide a basis for convenient analysis on the field, sprint mechanical variables may provide deeper insights into individual biomechanical limitations. The values presented here can be used by practitioners to develop individual training interventions.

## Supporting information

S1 File Excel File containing sample size, group means and SD for all variables across sports. (DOCX)
