# Supplementary material for: Sprint mechanical variables in elite athletes: Are force-velocity profiles sport specific or individual?
Source: PLoS One. 2019 Jul 24;14(7):e0215551. doi: 10.1371/journal.pone.0215551 (PMC6655540; doi:10.1371/journal.pone.0215551)
Supplement: S1 File — (DOCX) [file pone.0215551.s001.docx]

Mean values (± SD) of sprint mechanical outputs in Norwegian national team athletes.

| **Discipline (sex)** | ***N*** | **F0** | **V0** | **P0** | **S_FV_** | **RF** | **D_RF_** |
| --- | --- | --- | --- | --- | --- | --- | --- |
|  |  | *(N∙kg^-1^)* | *(m∙s^-1^)* | *(W∙kg^-1^)* | *(slope∙kg^-1^)* | *(%)* | *(%)* |
| Alpine skiing (W) | 10 | 7.7 ± 0.3 | 7.8 ± 0.4 | 14.9 ± 1.0 | -0.99 ± 0.06 | 42.6 ± 1.2 | -9.3 ± 0.6 |
| Alpine skiing (M) | 13 | 8.3 ± 0.5 | 9.0 ± 0.4 | 18.6 ± 1.2 | -0.92 ± 0.07 | 46.5 ± 1.3 | -8.6 ± 0.6 |
| Athletic jumping (W) | 8 | 7.8 ± 0.4 | 8.8 ± 0.4 | 17.2 ± 1.4 | -0.89 ± 0.06 | 45.1 ± 1.5 | -8.3 ± 0.5 |
| Athletic jumping (M) | 9 | 8.4 ± 0.5 | 10.0 ± 0.4 | 20.9 ± 1.3 | -0.85 ± 0.06 | 48.5 ± 1.2 | -7.8 ± 0.5 |
| Athletic sprinting (W) | 5 | 8.5 ± 0.3 | 9.2 ± 0.2 | 19.7 ± 0.7 | -0.93 ± 0.03 | 47.6 ± 0.7 | -8.5 ± 0.3 |
| Athletic sprinting (M) | 8 | 9.0 ± 0.4 | 10.3 ± 0.5 | 23.2 ± 1.2 | -0.87 ± 0.07 | 50.5 ± 1.0 | -7.9 ± 0.7 |
| Athletic throwing (W) | 10 | 7.7 ± 0.5 | 8.1 ± 0.4 | 15.6 ± 1.3 | -0.95 ± 0.08 | 43.4 ± 1.5 | -9.0 ± 0.8 |
| Athletic throwing (M) | 14 | 8.3 ± 0.6 | 9.1 ± 0.4 | 18.8 ± 1.8 | -0.91 ± 0.07 | 46.6 ± 1.8 | -8.5 ± 0.7 |
| Bandy (W) | 13 | 7.3 ± 0.6 | 7.3 ± 0.2 | 13.4 ± 1.3 | -1.00 ± 0.06 | 40.7 ± 1.7 | -9.5 ± 0.6 |
| Bandy (M) | 23 | 7.9 ± 0.6 | 8.7 ± 0.3 | 17.3 ± 1.4 | -0.91 ± 0.07 | 45.1 ± 1.5 | -8.5 ± 0.7 |
| Basket (M) | 10 | 8.3 ± 0.5 | 8.7 ± 0.4 | 18.0 ± 1.9 | -0.95 ± 0.04 | 45.9 ± 1.9 | -8.9 ± 0.4 |
| Beach-/volleyball (M) | 23 | 8.6 ± 0.4 | 8.6 ± 0.4 | 18.4 ± 1.3 | -1.01 ± 0.06 | 46.4 ± 1.3 | -9.4 ± 0.6 |
| Bobsleigh (M) | 9 | 9.1 ± 0.4 | 9.8 ± 0.2 | 22.3 ± 1.2 | -0.93 ± 0.05 | 49.9 ± 1.0 | -8.5 ± 0.4 |
| Combat sports (W) | 17 | 7.1 ± 0.4 | 7.4 ± 0.4 | 13.1 ± 1.2 | -0.97 ± 0.06 | 40.3 ± 1.5 | -9.2 ± 0.6 |
| Combat sports (M) | 32 | 8.1 ± 0.6 | 8.7 ± 0.4 | 17.6 ± 1.7 | -0.93 ± 0.08 | 45.4 ± 1.7 | -8.7 ± 0.8 |
| Cross-country skiing (W) | 8 | 7.3 ± 0.5 | 7.5 ± 0.6 | 13.7 ± 1.7 | -0.97 ± 0.07 | 41.0 ± 2.2 | -9.2 ± 0.7 |
| Cross-country skiing (M) | 15 | 7.9 ± 0.7 | 8.5 ± 0.6 | 16.8 ± 2.4 | -0.93 ± 0.06 | 44.6 ± 2.5 | -8.7 ± 0.6 |
| Fencing (W) | 5 | 6.9 ± 0.3 | 7.2 ± 0.4 | 12.3 ± 0.9 | -0.96 ± 0.06 | 39.4 ± 1.2 | -9.1 ± 0.6 |
| Fencing (M) | 10 | 7.8 ± 0.4 | 8.2 ± 0.3 | 16.0 ± 1.0 | -0.95 ± 0.06 | 43.9 ± 1.1 | -8.9 ± 0.5 |
| Handball (W) | 32 | 7.8 ± 0.5 | 8.2 ± 0.4 | 16.0 ± 1.6 | -0.94 ± 0.04 | 43.8 ± 1.8 | -8.9 ± 0.4 |
| Handball (M) | 18 | 8.4 ± 0.4 | 9.1 ± 0.4 | 19.0 ± 1.1 | -0.92 ± 0.06 | 46.9 ± 1.1 | -8.5 ± 0.6 |
| Icehockey (M) | 34 | 8.4 ± 0.6 | 9.0 ± 0.4 | 18.8 ± 1.7 | -0.93 ± 0.08 | 46.7 ± 1.7 | -8.6 ± 0.7 |
| Mogul skiing (W) | 5 | 7.6 ± 0.1 | 8.0 ± 0.4 | 15.2 ± 0.9 | -0.96 ± 0.05 | 43.0 ± 1.0 | -9.0 ± 0.5 |
| Mogul skiing (M) | 14 | 8.3 ± 0.4 | 8.8 ± 0.3 | 18.4 ± 0.9 | -0.95 ± 0.06 | 46.3 ± 0.9 | -8.8 ± 0.5 |
| Nordic combined (M) | 22 | 8.3 ± 0.5 | 8.9 ± 0.4 | 18.5 ± 1.3 | -0.94 ± 0.07 | 46.4 ± 1.3 | -8.8 ± 0.6 |
| Ski jumping (W) | 11 | 7.5 ± 0.4 | 7.6 ± 0.3 | 14.2 ± 0.7 | -1.00 ± 0.09 | 41.8 ± 0.9 | -9.5 ± 0.8 |
| Ski jumping (M) | 28 | 8.5 ± 0.5 | 8.7 ± 0.5 | 18.4 ± 1.8 | -0.97 ± 0.06 | 46.3 ± 1.8 | -9.0 ± 0.6 |
| Snowboard (W) | 5 | 7.7 ± 0.4 | 7.3 ± 0.4 | 14.0 ± 1.1 | -1.05 ± 0.07 | 41.5 ± 1.3 | -10.0 ± 0.7 |
| Snowboard (M) | 9 | 8.6 ± 0.5 | 8.6 ± 0.3 | 18.4 ± 1.4 | -1.00 ± 0.06 | 46.4 ± 1.3 | -9.3 ± 0.6 |
| Soccer (W) | 93 | 7.6 ± 0.5 | 8.1 ± 0.4 | 15.5 ± 1.3 | -0.94 ± 0.07 | 43.2 ± 1.5 | -8.9 ± 0.7 |
| Soccer (M) | 57 | 8.5 ± 0.5 | 9.2 ± 0.4 | 19.5 ± 1.4 | -0.92 ± 0.07 | 47.4 ± 1.4 | -8.5 ± 0.7 |
| Speed skating (W) | 12 | 7.5 ± 0.4 | 7.9 ± 0.3 | 14.9 ± 1.0 | -0.96 ± 0.06 | 42.5 ± 1.1 | -9.1 ± 0.6 |
| Speed skating (M) | 22 | 7.8 ± 0.6 | 8.9 ± 0.5 | 17.2 ± 1.6 | -0.88 ± 0.08 | 44.9 ± 1.7 | -8.2 ± 0.8 |
| Table tennis (M) | 13 | 8.0 ± 0.4 | 8.4 ± 0.5 | 16.7 ± 1.5 | -0.95 ± 0.07 | 44.5 ± 1.6 | -8.9 ± 0.7 |
| Telemark skiing (W) | 5 | 7.3 ± 0.6 | 7.5 ± 0.6 | 13.8 ± 2.2 | -0.97 ± 0.04 | 41.2 ± 2.8 | -9.2 ± 0.4 |
| Telemark skiing (M) | 13 | 8.1 ± 0.6 | 8.6 ± 0.3 | 17.4 ± 1.3 | -0.94 ± 0.07 | 45.4 ± 1.4 | -8.8 ± 0.7 |
| Tennis (W) | 7 | 7.4 ± 0.4 | 7.5 ± 0.4 | 13.9 ± 1.2 | -0.98 ± 0.05 | 41.4 ± 1.5 | -9.3 ± 0.5 |
| Tennis (M) | 11 | 8.2 ± 0.4 | 8.7 ± 0.3 | 17.8 ± 1.1 | -0.94 ± 0.04 | 45.7 ± 1.2 | -8.8 ± 0.4 |
| Weight-/powerlifting (M) | 13 | 8.0 ± 0.5 | 8.3 ± 0.5 | 16.7 ± 1.5 | -0.97 ± 0.08 | 44.5 ± 1.6 | -9.1 ± 0.7 |

W = women, M = men, P0 = maximal horizontal power, F0 = maximal horizontal force, V0 = theoretical maximal velocity, S_FV_ = force-velocity slope, RF = ratio of force, D_RF_ = index of force application technique.
